# Author Correction: Search and rescue at sea aided by hidden flow structures

**DOI:** 10.1038/s41467-020-16955-6

**Published:** 2020-06-25

**Authors:** Mattia Serra, Pratik Sathe, Irina Rypina, Anthony Kirincich, Shane D. Ross, Pierre Lermusiaux, Arthur Allen, Thomas Peacock, George Haller

**Affiliations:** 1 School of Engineering and Applied Sciences, Harvard University, Cambridge, MA 02138 USA; 2 Department of Physics and Astronomy, University of California, Los Angeles, CA 90095 USA; 3 Physical Oceanography Department, Woods Hole Oceanographic Institution, Woods Hole, MA 02543 USA; 4 Aerospace and Ocean Engineering, Virginia Tech, Blacksburg, VA 24061 USA; 5 Mechanical Engineering Dep., Massachusetts Institute of Technology, Cambridge, MA 02139 USA; 6 U.S. Coast Guard Office of Search and Rescue, Washington, DC 20593 USA; 7 Institute for Mechanical Systems, ETH Zürich, 8092 Zurich, Switzerland

**Keywords:** Natural hazards, Physical oceanography, Applied mathematics, Fluid dynamics

Correction to: *Nature Communications* 10.1038/s41467-020-16281-x, published online 26 May 2020.

The original version of this Article contained an incorrect hyperlink in reference 17. This has now been corrected in the PDF and HTML versions of the Article.

