# Supplementary material for: Search and rescue at sea aided by hidden flow structures
Source: Nat Commun. 2020 May 26;11:2525. doi: 10.1038/s41467-020-16281-x (PMC7250873; doi:10.1038/s41467-020-16281-x)
Supplement: Supplementary file 1 — Supplementary Information [file 41467_2020_16281_MOESM1_ESM.pdf]

Supplementary Information
Search and rescue at sea aided by hidden flow structures

Serra et al.

Supplementary Methods

TRAPs and uncertainties

The partial differential equations generating the fluid velocities used in SAR account for a broad set of uncertainties[1;2]. These affect the initial conditions of all state variables including velocity, as well as external forcing and boundary conditions such as tidal forcing, atmospheric forcing fluxes, lateral boundary conditions, etc. (see Model velocity dataset). With a set of ensemble velocities at hand, typical Lagrangian methods used in SAR compute their corresponding trajectories with the last seen location and time used as initial conditions. Here we consider a total of 9 ensemble forecast velocities modelling part of the above uncertainties during the 2018 field experiment shown in Fig. 6, and compute trajectories of the different ensemble velocities from the drifter and manikin release locations (Fig. 1). Supplementary Figure 1a shows the drifter and manikin trajectories, initially released at point B, along with fluid particle trajectoties of the ensemble velocities (gray) and the model-based TRAP computed from the center-forecast velocity. Even within two hours from the release, ensemble trajectories already show visible differences within each other and with the actual drifter and manikin trajectories, yet all converge towards nearby TRAPs. Supplementary Figure 1b shows the same as Supplementary Fig. 1a for all the release location of our 2018 experiment.

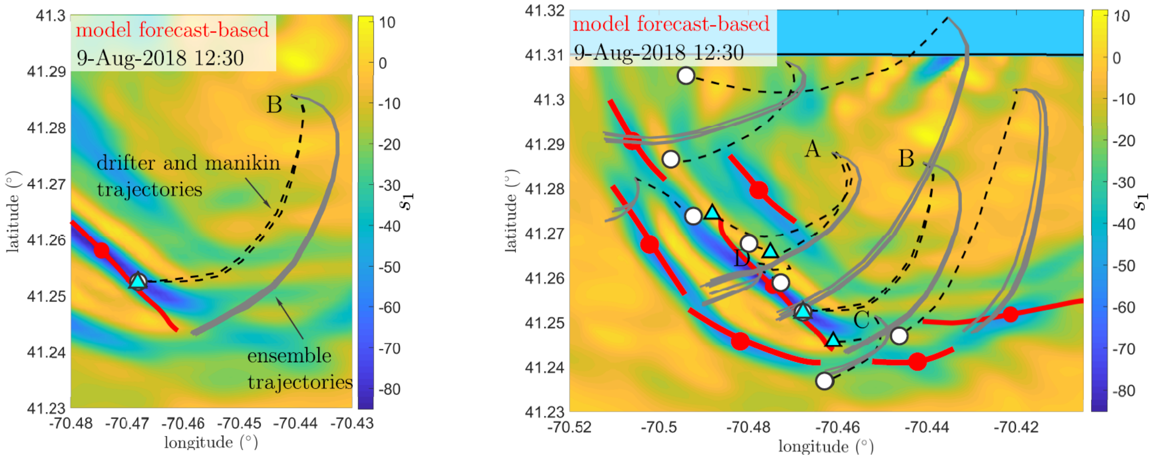

(a) (b)

Supplementary Figure 1: Robustness of TRAPs under uncertainties. (a) Drifter and manikin trajectories (dashed lines) released at point B during the field experiment on the 9th of August 2018. Grey lines show trajectories of fluid particles from nine different model based ensemble velocities accounting for uncertainties (see Model velocity dataset). The TRAP is computed from the center-forecast model velocity. (b) Same as (a) for all release locations in the 2018 experiment described in Fig. 6. Despite trajectories from model velocity realizations and real drifters and manikins are visibly different within a few hours, they converge to nearby TRAPs.

To gain insights about the predictive power of TRAPs and Lagrangian methods under uncertainties, one can encode the above velocity field uncertainties in the stochastic ordinary differential equation for particle motions $d\mathbf{x}(t) = \mathbf{v}(\mathbf{x}(t), t)dt + \mathbf{R}(\mathbf{x}(t), t)d\mathbf{W}(t)$ where $\mathbf{x}(t)$ is the random position vector, $\mathbf{v}(\mathbf{x}(t), t)$ is the deterministic drift velocity and $\mathbf{W}(t)$ is a two-dimensional Weiner process with diffusion matrix $\mathbf{R}(\mathbf{x}(t), t)$. Then, the uncertain rate-of-strain tensor is

$\mathbf{S}_u(\mathbf{x},t) = \mathbf{S}(\mathbf{x},t) + \mathbf{S}_R(\mathbf{x},t)d\mathbf{W}(t)$, where $\mathbf{S}_R(\mathbf{x},t) = \mathrm{sym}(\partial_x\mathbf{R}(\mathbf{x}(t),t) + \partial_y\mathbf{R}(\mathbf{x}(t),t))$. In the simplest case of spatially homogeneous uncertainties $\mathbf{S}_R(\mathbf{x},t) = \mathbf{0}$, hence TRAP predictions remain unaffected while Lagrangian (trajectory based) predictions will have inherent errors that grow both with the largest Lyapunov exponent and $\sqrt{t}$. For TRAPs to be significantly affected by uncertainties, their spatial inhomogeneities should be comparable to $\mathbf{S}(\mathbf{x},t)$, which means the model is highly inaccurate. These simple considerations suggest that TRAPs are intrinsically robust predictors under uncertainties over short times.

TRAPs and velocity field divergence

By Liouville's theorem[3], the infinitesimal phase-space volume of a dynamical system shrinks along a trajectory as long as the trajectory is contained in a domain of negative divergence. Guckenheimer and Holmes[4] conclude that if a steady vector field points everywhere inwards along the boundary of a compact region of negative divergence, then the region contains a nonempty attractor. This criterion, however, will never hold in an unsteady flow on its extended phase space of positions and time. Consequently, there is no applicable, classical dynamical systems technique to find attractors based on the velocity-field divergence in a general, non-autonomous system. This gap has been filled by the variational theory LCS[5] for finite-time flows, and OECS[6], as its instantaneous limit. Indeed, accumulation of particles along lines for longer time intervals is well known to happen along attracting LCS in incompressible velocity fields[7;8]. Our results show that even regions of positive divergence can contain curves that collect drifters/manikins.

The attraction of TRAPs arises from the combination of isotropic and anisotropic deformations (Supplementary Figure 2a). The isotropic component is due to the instantaneous divergence of the velocity field ($\nabla\cdot\mathbf{v}$), while the anisotropic one to shear, both of which are encoded in $\mathbf{S}$, and thus in the definition of TRAPs. Supplementary Figures 2b-c illustrate short-term clustering

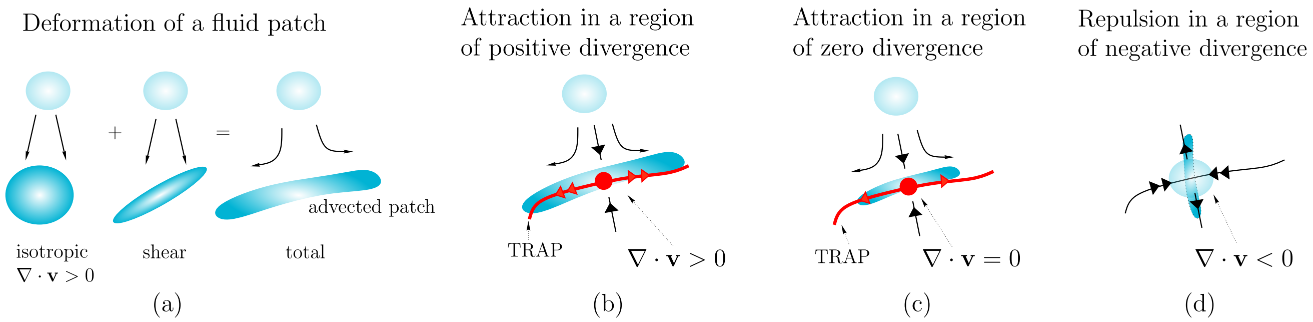

Supplementary Figure 2: TRAPs vs horizontal divergence. (a) At leading order, the short-term deformation of a fluid patch arises from an isotropic contribution quantified by the divergence of the velocity field ($\nabla\cdot\mathbf{v}$), and an isotropic contribution due to shear. Because of this combination, short-term attraction and clustering can occur in regions of positive divergence (b) or zero divergence (c), invariably captured by TRAPs (red). (d) Tracers escape a region of negative divergence.

regions, correctly predicted by TRAPs, where the local divergence is positive or zero. Panel b is similar to Fig. 4c, panel c to Fig. 2b or to the findings in Ref.[6]. Supplementary Figure 2d shows that tracers can escape a region of negative divergence.

High frequency radar velocity dataset

The WHOI high frequency radar system (HFR), as operated during the 2014-2017 experiments, consisted of 3 land-based sites spaced at 10km intervals along the south side of Martha's Vineyard, MA. These 25MHz systems were run using a combination of 350kHz transmit bandwidth and low transmit power (10W max) which allowed all systems to achieve resolutions of 429m and ranges of 30km (see[9] for details). Doppler spectra received from each system were processed using advanced methods[10] into radial velocity estimates every 15min based on a 24min averaging window. Radial velocity estimates were quality controlled (QC) before inclusion into the vector velocity estimates using standard time-series QC techniques. These data were combined into vector velocities on a uniform 800m resolution grid, given in latitude and longitude coordinates, using a unique weighted least squares technique that employed non-velocity based signal quality metrics to weight the data to increase the accuracy of the final product. Two successive estimates of the 15min radials are used to estimate the vector (east and north) velocities on a 1/2h time interval centered on the hour. The spatial extent of the vector velocities was limited by theoretical Geometrical Dilution of Precision (GDOP) values less than 1.75. An error estimate for the east, north, and the norm of the vector velocity components is given. This estimate uses the radial velocity error estimates (the weighted standard deviation of the individual HF radar radial returns found within each 5 degree azimuthal bin average) in a standard vector error calculation [11;12;13].

Because of occasional measurement deficiency, there are grid points at which the velocity is not available within the region of interest (blue dots inside the dashed curve in Supplementary Fig. 3a). To overcome this limitation, we devise a simple interpolation scheme by which we can obtain velocity everywhere within a well-defined boundary. Specifically, we first compute the boundary of this region (the dashed curve in Supplementary Fig. 3), using the Delaunay triangulation function in MATLAB. For each time instance at which the velocity field data is available, we obtain an estimate of the velocity at the blue points inside the boundary using a linear scattered interpolation scheme (see Supplementary Fig. 3b). Once the velocity field is available within the black boundary, we convert it from its original units of ms^{-1} to degrees per day.

Finally, we note that HFR velocities are computed by averaging the raw radial velocity estimates with a 800m window radius at each grid point, spaced 800m apart from each other[9]. To yield an accurate computation of TRAPs from HFR velocities consistent with the way the data are processed, we smooth $\nabla \mathbf{v}(\mathbf{x}, t)$ with a spatial average filter whose width corresponds to two grid sizes (1600m), as in Ref.[14].

Finite-size domain

When the velocity field is available over a finite-sized domain, as the case of HFR velocity, Lagrangian Coherent Structures (LCSs) methods[5;15] invariably provide incomplete coverage because of particles leaving the domain. Even if the velocity field is derived from models, they typically assimilate in situ measurements[16] to enhance model predictions in a specific region of interest, making the resulting velocity field more accurate in a finite-size domain. Assuming that such a region is the domain over which HFR velocity is available, Supplementary Fig. 4 shows the coverage reduction of LCSs methods for two different integration times. We compute fluid trajectories by integrating the HFR velocity field starting on the 4th of August 2014 at 8pm, with a dense

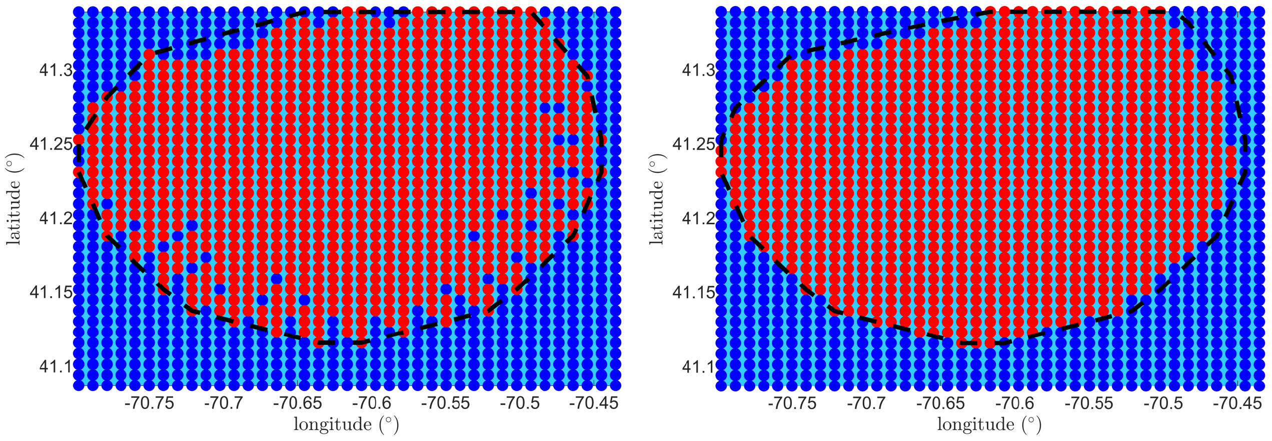

(a) (b)

Supplementary Figure 3: HFR velocity field coverage. Latitude-longitude grid points where the velocity values are available at a particular time are shown in red. Even within the region of interest bounded by the dashed line, there are blue points where the velocity field is not available due to occasional measurement deficiency. The dashed curve is the convex hull enclosing all the red points. Velocity field grid points (a) before and (b) after interpolation.

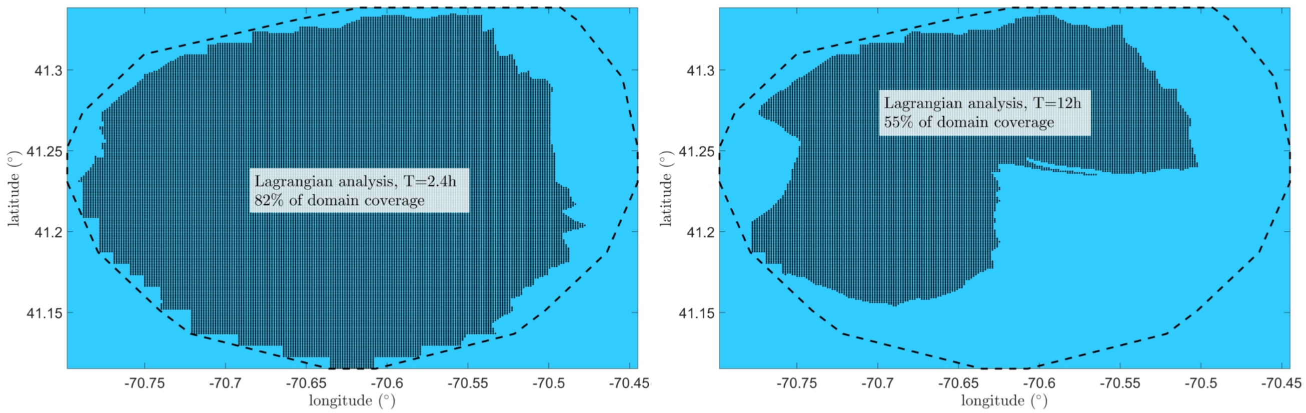

(a) (b)

Supplementary Figure 4: Effect of finite-size domains on Lagrangian methods. Integrating the HFR velocity field starting at 4th of August 2014 at 8pm, we compute trajectories of fluid particles from a dense set of initial conditions covering the entire domain bounded by the dashed curve. (a) After 2.4h, 82% of fluid particles (black points) are within the finite-size domain. (b) After 12h, only 55% of fluid particles are within the finite-size domain.

set of initial conditions covering the entire domain bounded by the dashed curve. After 2.4h (12h) any Lagrangian method can provide information only on 82% (55%) of the region where the velocity field is available. Eulerian methods, instead, provide full coverage of the domain because they do not rely on particle trajectories.

In a SAR operation, the extent of the search domain evolves over time, starting from the last seen location area and its associated uncertainties. The domain then grows according to the overall Lagrangian transport and its total extent is only constrained by the shoreline of the body of water where the case occurs. TRAPs, therefore, can be computed within this evolving domain,

readily providing regularly updated optimal search paths. Finally, we note that the test area off Martha Vineyard analyzed here would be typical for a first-day search.

Model velocity dataset

We use the MIT Multidisciplinary Simulation, Estimation, and Assimilation Systems (MIT-MSEAS)[17;18] Primitive-Equation (PE) ocean modeling system to compute ocean surface velocity forecasts in the Nantucket and Martha's Vineyard coastal region during August 2017 and 2018. The modeling system is set-up in an implicit 2-way nesting configuration, and provided forecasts of the ocean state variable fields (three-dimensional velocity, temperature, salinity, and sea surface height) every hour with a spatial resolution of 200m in the Martha's Vineyard domain and of 600m in the larger Shelf domain. The ocean forecasts are initialized using historical and synoptic ocean CTD data from the National Marine Fisheries Service (NMFS) and the Martha's Vineyard Coastal Observatory (MVCO), SST images from the Johns Hopkins University's Applied Physics Lab (JHU APL), and other data from available sources. These ocean simulations are forced by atmospheric flux fields forecast by the National Centers for Environmental Prediction (NCEP) and tidal forcing from TPXO8, but adapted to the high-resolution bathymetry and coastlines[19]. Finally, in situ CTD measurement have been assimilated into the modeling system at the start of the experiment within the first few days of August.

The deterministic 2-way nesting ocean forecast initialized from the estimated ocean state conditions at a particular time is referred to as the central forecast. The ensemble forecasts were initialized using Error Subspace Statistical Estimation procedures[20]. The forecasts within the ensemble were commonly initialized from perturbed initial conditions of all state variables (temperature, salinity, velocity, sea surface height) and forced by perturbed tidal forcing, atmospheric forcing fluxes and lateral boundary conditions. These initial, forcing and boundary perturbations are created so as to represent the expected uncertainties in each of these quantities, respecting ocean physics and in accord with the few observed data misfits. Each ensemble members were 2-way nested in two domains and required respecting domain embedding conditions. Finally, parameter uncertainties (bottom drag, mixing coefficients, etc.) were also modeled by perturbing the values of parameters for each ensemble forecast.

Ensemble forecasts were issued twice daily during the 2 weeks of the 2017 and 2018 experiment (see http://mseas.mit.edu/Sea_exercises/NSF_ALPHA/2018/), with the number of ensemble forecasts issued varying between 7 and 100 depending on the number of computing units available and on computational power. The nine 2018 MSEAS ensemble forecasts utilized for the present study correspond to parametric uncertainties only, representing uncertainties in the surface wind mixing, tidal mixing and tidal bottom friction.

Drifter dataset

The drifters used in our experiments (Fig. 3) have technical specifications similar to the original CODE drifters designed by Dr. Russ Davis of the Scripps Institution of Oceanography. Each drifter consists of a thin (15cm in diameter) 1m long cylindrical metal body with 0.5m long metal foldable cross-shaped upper arms and lower legs that held a rectangular cloth sail. The drifter is attached by four 20cm rope segments to 4 small (15cm in diameter) round plastic surface buoys for floatation. The round buoy shape minimizes the wave effects compared to flat buoys, the ropes minimizes tilting of the sail compared to the "solid-neck" drifter design, and the large

body-to-buoy size ratio insures good water-following characteristics. The drifters are equipped with GPS transmitters that provide positioning fixes every 5min. Based on land tests conducted prior to deployment, the STD of the GPS positioning error is on the order of a few meters (exact values depend on the sky view and location). Estimates of the expected wind slippage of CODE type drifters with standard sails such as ours are 1–2 cm/s in light wind conditions similar to those during our field experiment[21;22]. Drifters of the same design are routinely used by the U.S. Coast Guard in SAR operations, as well as in our previous field experiments south of Martha's Vineyard, MA[23;24].

Manikin dataset

We used OSCAR Water Rescue Training manikins (Fig. 3) manufactured by Emerald Marine Products (Edmonds, WA) for man-overboard rescue training. Each manikin consists of eight heavy-duty vinyl body parts, PVC fill/drain fittings, six stainless steel joints and two galvanized lifting shackles. The manikin filled with water replicates an 82 kg rescue victim, 1.83 m tall and 0.46 m wide (chest). For an accurate simulation of a person in water, the manikin is filled with water to float at chest level. The manikins are equipped with the same GPS transmitters used for drifters, which provide positioning fixes every 5min.

Supplementary Note 1

Computational times in SAR

Here we provide a rough estimate of the computational time gain of TRAP-based SAR planning compared to existing techniques. A Search and Rescue Optimal Planning System (SAROPS) consists of approximately twelve steps[25]. About 70% of the total computational time is required for downloading the data from the Environmental Data Server (EDS) to the SAROPS computers and for generating search patterns from the probability-distribution maps for the lost object's location. High-performance SAROPS computers are mainly needed to perform Monte Carlo simulations and for computing search paths from the 2D probability maps. The robustness under uncertainties and the Eulerian nature of TRAPs, which are one-dimensional curves suitable for search-asset allocation, eliminate the need for Monte Carlo simulations and further post-processing of search patterns. TRAPs, therefore, could be potentially computed at the EDS-level, reducing the computational time by at least 50%, or at least providing valuable additional information to existing strategies at no extra costs.

References

[1] P. F. J. Lermusiaux, "Uncertainty estimation and prediction for interdisciplinary ocean dynamics," *Journal of Computational Physics*, vol. 217, no. 1, pp. 176–199, 2006.

[2] P. F. J. Lermusiaux, C.-S. Chiu, G. G. Gawarkiewicz, P. Abbot, A. R. Robinson, R. N. Miller, P. J. Haley, Jr, W. G. Leslie, S. J. Majumdar, A. Pang, and F. Lekien, "Quantifying uncertainties in ocean predictions," *Oceanography*, vol. 19, no. 1, pp. 92–105, 2006.

[3] V. Arnold, *Ordinary Differential Equations.* MIT Press, Boston, 1973.

[4] J. Guckenheimer and P. Holmes, *Nonlinear oscillations, dynamical systems, and bifurcations of vector fields*, vol. 42. Springer Science & Business Media, 1983.

[5] G. Haller, "Lagrangian coherent structures," *Annual Rev. Fluid. Mech*, vol. 47, pp. 137–162, 2015.

[6] M. Serra and G. Haller, "Objective Eulerian coherent structures," *Chaos*, vol. 26, no. 5, p. 053110, 2016.

[7] F. Beron-Vera, M. Olascoaga, and G. Goni, "Oceanic mesoscale vortices as revealed by Lagrangian coherent structures," *Geophys. Res. Lett.*, vol. 35, p. L12603, 2008.

[8] M. Olascoaga, F. Beron-Vera, G. Haller, J. Trinanes, M. Iskandarani, E. Coelho, B. Haus, H. Huntley, G. Jacobs, A. Kirwan, *et al.*, "Drifter motion in the Gulf of Mexico constrained by altimetric Lagrangian coherent structures," *Geophys. Res. Lett.*, vol. 40, no. 23, pp. 6171–6175, 2013.

[9] A. Kirincich, "Remote sensing of the surface wind field over the coastal ocean via direct calibration of HF radar backscatter power," *J. Atmosph. and Oceanic Tech.*, vol. 33, no. 7, pp. 1377–1392, 2016.

[10] A. Kirincich, T. De Paolo, and E. Terrill, "Improving HF radar estimates of surface currents using signal quality metrics, with application to the MVCO high-resolution radar system," *J. Atmos. Oceanic Technol.*, vol. 29, no. 9, pp. 1377–1390, 2012.

[11] D. Wells, N. Beck, D. Delikaraoglou, A. Kleusberg, E. Krakiwsky, G. Lachapelle, R. Langley, M. Nakiboglu, K. Schwarz, J. Tranquilla, *et al.*, "Guide to gps positioning, 2nd printing with corrections," *Canadian GPS Associates, Fredericton NB*, 1987.

[12] R. D. Chapman and H. C. Graber, "Validation of hf radar measurements," *Oceanography*, vol. 10, no. 2, pp. 76–79, 1997.

[13] B. Lipa, "Uncertainties in seasonde current velocities," in *Proceedings of the IEEE/OES Seventh Working Conference on Current Measurement Technology, 2003.*, pp. 95–100, IEEE, 2003.

[14] A. Kirincich, "The occurrence, drivers, and implications of submesoscale eddies on the Martha's Vineyard inner shelf," *J. Phys. Oceanogr.*, vol. 46, no. 9, pp. 2645–2662, 2016.

[15] A. Hadjighasem, M. Farazmand, D. Blazevski, G. Froyland, and G. Haller, "A critical comparison of Lagrangian methods for coherent structure detection," *Chaos*, vol. 27, no. 5, p. 053104, 2017.

[16] A. Allen, "Performance of GPS/Argos self-locating datum marker buoys (SLDMBs)," in *OCEANS'96. MTS/IEEE. Prospects for the 21st Century. Conference Proceedings*, vol. 2, pp. 857–861, IEEE, 1996.

[17] P. Haley and P. Lermusiaux, “Multiscale two-way embedding schemes for free-surface primitive equations in the Multidisciplinary Simulation, Estimation and Assimilation System,” *Ocean Dyn*, vol. 60, no. 6, pp. 1497–1537, 2010.

[18] P. J. Haley, Jr., A. Agarwal, and P. F. J. Lermusiaux, “Optimizing velocities and transports for complex coastal regions and archipelagos,” *Ocean Modeling*, vol. 89, pp. 1–28, 2015.

[19] O. G. Logutov and P. F. J. Lermusiaux, “Inverse barotropic tidal estimation for regional ocean applications,” *Ocean Modelling*, vol. 25, no. 1–2, pp. 17–34, 2008.

[20] P. F. J. Lermusiaux, “On the mapping of multivariate geophysical fields: Sensitivities to size, scales, and dynamics,” *Journal of Atmospheric and Oceanic Technology*, vol. 19, no. 10, pp. 1602–1637, 2002.

[21] C. Ohlmann, P. White, L. Washburn, B. Emery, E. Terrill, and M. Otero, “Interpretation of coastal HF radar–derived surface currents with high-resolution drifter data,” *J. Atmos. Oceanic Technol.*, vol. 24, no. 4, pp. 666–680, 2007.

[22] R. Poulain, P.and Gerin, E. Mauri, and R. Pennel, “Wind effects on drogued and undrogued drifters in the eastern Mediterranean,” *J. Atmos. Oceanic Technol.*, vol. 26, no. 6, pp. 1144–1156, 2009.

[23] I. Rypina, A. Kirincich, and I. Limeburner, R.and Udovydchenkov, “Eulerian and Lagrangian correspondence of high-frequency radar and surface drifter data: Effects of radar resolution and flow components,” *J. Atm. and Oceanic Tech.*, vol. 31, no. 4, pp. 945–966, 2014.

[24] I. Rypina, A. Kirincich, S. Lentz, and M. Sundermeyer, “Investigating the eddy diffusivity concept in the coastal ocean,” *J. Phys. Oceangr.*, vol. 46, no. 7, pp. 2201–2218, 2016.

[25] United States Coast Guard, “International aeronautical and maritime search and rescue manual,” tech. rep., US Coast Guard, 2013.
