# Supplementary material for: Search and rescue at sea aided by hidden flow structures
Source: Nat Commun. 2020 May 26;11:2525. doi: 10.1038/s41467-020-16281-x (PMC7250873; doi:10.1038/s41467-020-16281-x)
Supplement: Supplementary file 3 — Description of Additional Supplementary Files [file 41467_2020_16281_MOESM3_ESM.pdf]

Description of Additional Supplementary Files

File Name: Supplementary Movie 1

Description: Time evolution movie associated with Figs. 4a-c.
